# Injectable contraception provided by community-based health workers: one important step toward meeting unmet need

**DOI:** 10.9745/GHSP-D-13-00152

**Published:** 2013-11-14

## Abstract

Community-based provision of injectable contraception continues to advance and is gaining wider acceptance—a major step toward meeting unmet need. However, fully addressing family planning need will require access to a much wider range of methods, including long-acting reversible contraception and permanent methods.

Following the endorsement by a technical consultation supported by the World Health Organization (WHO) in 2009,^1^ a number of countries have embraced provision of injectable contraception by community-based health workers (CHWs). Two articles in this issue of GHSP support such provision and increase our understanding of it.

In rural Kenya, Olawo et al.^2^ document the safety and acceptability of, and marked increase in, the use of injectables with CHW provision. And their efforts contributed importantly toward approval of CHW provision by the Kenyan Ministry of Health. Chin-Quee and colleagues^3^ found similar results in Zambia, and they determined that if CHWs are already deployed, adding injectables to their work appears cost-effective. A major advantage of such community-based provision is that it supports equity by improving contraceptive choice for rural and low-income women with few contraceptive options.

Still, while CHW provision of injectables is an important and sensible advance that should be widely expanded, it has important challenges relating to coverage provided by CHWs and optimum contraceptive method choice. Regarding coverage, a recent constructive initiative has called for 1 million community health workers in sub-Saharan Africa by 2015.^4^ Yet for now, with some notable exceptions, coverage of community-based service delivery for health services in low- and middle-income countries is rather limited. And, as documented by Hodgins and colleagues,^5^ relatively few caregivers access key child health services from CHWs. Regarding optimum choice, although CHWs are clearly able to provide the short-acting methods of condoms, pills, and injectables (as well as the Standard Days Method®, the Lactational Amenorrhea Method (LAM), and emergency contraceptive pills), women and couples should have a much wider selection.

Injectable contraceptives have become the dominant method in many developing countries, especially in Africa.^6^ They have features that are attractive to many women, and they are also relatively easy to deliver and are appealing to providers. But demand for limiting additional pregnancies is rising rapidly in Africa,^7^ and continuation rates with all the short-acting methods are typically rather short. Expanding access to injectables is one excellent step toward meeting demand for family planning. But to make serious strides in meeting unmet need, expanding contraceptive choice, and advancing toward Family Planning 2020 goals,^8^ we must energetically support additional service delivery models to expand access to a wider range of methods—especially the long-acting reversible contraception (LARC) methods of implants and IUDs, as well as permanent methods of contraception. *–Global Health: Science and Practice*
